# An ethical advantage of autistic employees in the workplace

**DOI:** 10.3389/fpsyg.2024.1364691

**Published:** 2024-03-14

**Authors:** Lorne Hartman, Braxton Hartman

**Affiliations:** ^1^Department of Psychology, York University, Toronto, ON, Canada; ^2^Schulich School of Business, York University, Toronto, ON, Canada; ^3^Temerty Faculty of Medicine, University of Toronto, Toronto, ON, Canada

**Keywords:** autism, bystander effect, moral disengagement, neurodiversity, unethical behavior, workplace

## Abstract

Differences between autistic and nonautistic people are often framed as deficits. This research considers whether some of these differences might actually be strengths. In particular, autistic people tend to be less sensitive to their social environment than nonautistic people who are easily influenced by the judgments, opinions, beliefs and actions of others. Because autistic people are less susceptible to social influence, as employees they are more likely to take action when they witness an operational inefficiency or an ethical problem in the organization. By reporting problems, autistic employees may contribute to the introduction of innovations and improvements in organizational processes and effectiveness that result in superior performance. This paper considers whether and the extent to which these differences between autistic and nonautistic employees are moderated by “moral disengagement,” a set of interrelated cognitive mechanisms that allow people to make unethical decisions by deactivating moral self-regulatory processes. While previous research has shown that moral disengagement is related to unethical decisions, there is no research on whether and the extent to which autistic people are vulnerable to moral disengagement. Thirty-three autistic employees and 34 nonautistic employees completed an on-line survey to determine whether differences between autistic and nonautistic employees with regards to (1) likelihood they would voice concerns about organizational dysfunctions, and (2) degree to which they were influenced by the presence of others when deciding to intervene, are moderated by individual differences in moral disengagement. As predicted, autistic participants scored lower on moral disengagement than nonautistic participants. In terms of the moderating effects of moral disengagement, the results are mixed. Although moral disengagement reduced intervention likelihood, there was not a difference between autistic and nonautistic employees in the degree to which intervention likelihood was changed by an individual’s level of moral disengagement. However, there was a difference between autistic and nonautistic employees in the extent to which acknowledging the influence of others was affected by moral disengagement. These findings suggest that autistic adults are not just more likely to intervene when they witness dysfunction or misconduct in an organizational context; they are also less likely to engage in unethical behavior in general due to lower levels of moral disengagement. The reduced susceptibility to the bystander effect evidenced by autistic adults in the workplace may be accounted for, in part, by their lower levels of moral disengagement compared with nonautistic adults.

## Introduction

“*The only thing necessary for the triumph of evil is for good men to do nothing*.” This quote traces back to the utilitarian philosopher John Stuart Mill in an inaugural address at the University of St. Andrews in 1867. A century later, Darley and Latane (1968) published their findings regarding the influence of group inhibition on bystander intervention in emergencies. The “bystander effect” describes the tendency for people to be less inclined to assist others when in a group compared to when alone (see Fischer et al., 2011 for a comprehensive review). Three primary mechanisms account for this behavior (Ross and Nisbett, 2011): (1) “evaluation apprehension” where bystanders fear they may have misunderstood the situation and there is no need to help, (2) “pluralistic ignorance” which arises from relying on others’ reactions to gage the need for assistance, and (3) “diffusion of responsibility” which refers to a reduced feeling of responsibility to help when others are present.

The bystander effect and the causal mechanisms that mediate the effect have been linked to ethical misbehavior in organizations (e.g., Bazerman, 2022), that is behavior within organizations that causes direct harm to another individual or that violates widely accepted moral norms in society. While employees at times engage in unethical acts to benefit themselves, most corporate corruption scandals involve unethical acts that seek to benefit the organization (Umphress et al., 2010). Recent examples include the Boeing 737 Max fraud conspiracy, the Purdue Pharma opioid crisis, the Siemens bribery scandal, and Volkswagen’s “diesel-gate.” In all these cases, many individuals (hundreds) participated in the unethical acts, and an even larger number (thousands) were aware of the unethical behavior yet did nothing to stop it (Bazerman, 2022). Recently, it has been reported that autistic employees are more likely to do something or say something when they see something wrong happening in the organization (Hartman et al., 2023). By reporting problems, autistic employees may contribute to the introduction of innovations and improvements in organizational processes and effectiveness that result in superior performance. This paper considers whether and the extent to which these differences between autistic and nonautistic employees are moderated by moral disengagement.

Moral disengagement is a process that enables individuals to convince themselves that ethical standards do not apply in a particular context (Bandura, 2002). This is done by separating moral reactions from improper conduct, thus disabling the mechanism of self-condemnation -- a process of cognitively reconstruing or reframing bad behavior as being morally acceptable without changing the behavior or the moral standards. Bandura (2002) identified eight moral disengagement mechanisms representing a coherent set of cognitive tendencies that influence the way individuals make ethical decisions: (1) “moral justification” involves reframing unethical acts as serving a greater good, (2) “euphemistic labeling” uses sanitized language to rename harmful actions, making them appear less harmful, (3) “advantageous comparison” exploits the contrast between a considered behavior and an even more reprehensible one, making the former seem less severe, (4) “displacement of responsibility” involves attributing responsibility for one’s actions to authority figures who have tacitly condoned or explicitly directed those actions, (5) “diffusion of responsibility” spreads accountability for actions across members of a group, (6) “distortion of consequences” minimizes the seriousness of the effects of one’s actions, (7) “dehumanization” involves portraying the victims of one’s actions as undeserving of basic human considerations, and (8) “attribution of blame” assigns responsibility for the outcomes to the victims themselves, suggesting they deserve what happened to them. When people behave in ways that are inconsistent with their own personal values, moral disengagement prevents the activation of self-sanctions and self-condemnation, allowing for a wider range of behavior given the same moral standard. While previous research has shown that moral disengagement is positively related to unethical decisions (e.g., Detert et al., 2008), there is no research on whether and the extent to which autistic individuals are vulnerable to moral disengagement. This paper uses identity-first language to reflect the preferences of the autism community (Kenny et al., 2016).

However, prior research does suggest that autistic individuals are less vulnerable to cognitive biases (see Rozenkrantz et al., 2021 for a review). This difference could stem from their early experiences as young children, during which they did not learn the culturally supplied explanations for behavior typically acquired during this crucial period of neuroplasticity (Buon et al., 2013). As a result, in comparison to nonautistic controls, autistic participants are (1) less affected by the “sunk cost” bias, i.e., they are more likely to consider only current and future costs (avoidable costs) rather than irrecoverable (sunk) costs when making decisions (Fujino et al., 2017; Rogge, 2021), (2) less susceptible to “framing effects,” i.e., they are less likely to favor one of two mathematically identical options because of the way they are framed as a gain versus loss (De Martino et al., 2008; Shah et al., 2016), (3) more deliberative and less intuitive or emotional when reasoning (Brosnan et al., 2014; Levin et al., 2015; Brosnan et al., 2016; Farmer et al., 2017; Brosnan and Ashwin, 2023), (4) less susceptible to the “conjunction fallacy,” i.e., the tendency to favor multiple specific conditions over a single underlying cause because specific conditions seem more probable due to the salience of representative information (Morsanyi et al., 2010), (5) less likely to make moral judgments about an action based on an analysis of a person’s intention or character and more likely to rely on negative outcomes of the action (Moran et al., 2011; Komeda et al., 2016), (6) less susceptible to social influence and reputation management (Frith and Frith, 2011; Izuma et al., 2011), (7) less biased when updating self-referential beliefs (Kuzmanovic et al., 2019), (8) more likely to accept offers that are considered unfair but economically beneficial (Wang et al., 2019; Jin et al., 2020); (9) less susceptible to implicit bias based on race and gender (Kirchner et al., 2012; Birmingham et al., 2015); and (10) less likely to espouse false beliefs as bystanders about whether they were influenced by the presence of others (Hartman et al., 2023). Taken together, these studies suggest that autistic individuals are less susceptible to cognitive biases and exhibit more rational and bias-free processing of information which may be an advantage in ethical decision making.

The present study, to our knowledge, is the first to explicitly compare autistic and nonautistic adults on measures of moral disengagement. We predict that moral disengagement scores will be lower in autistic than nonautistic participants, and that variations in the level of moral disengagement will moderate previously reported differences between autistic and nonautistic employees with regards to susceptibility to the bystander effect (Hartman et al., 2023).

## Methods

### Participants

Survey data were collected form 33 autistic employees (average age = 36.12 years; 10 males and 23 females, i.e., 30% male and 70% female; average length of employment = 1 to 5 years, mean AQ score = 3.9) and 34 nonautistic employees (average age = 22.53 years; 18 males and 16 females, i.e., 53% male and 47% female; average length of employment = 1 to 5 years; mean AQ score = 2.2). Further detail on the demographic characteristics of participants is provided in the Appendix A; Supplementary Figures S1–S5.

### Instruments

Participants completed the 10-item version of the AQ or *Autism Spectrum Quotient* (Lundqvist and Lindner, 2017) to confirm diagnosis. Items were assessed on a 5-point scale ranging from 1 (*strongly disagree*) to 5 (*strongly agree*). A sample item from this scale is “I find it easy to do more than one thing at once.” Participants also completed an 8-item version of a *Moral Disengagement Survey* similar to one used in multiple studies with adults (Detert et al., 2008). Items were assessed on a 5-point scale ranging from 1 (*strongly disagree*) to 5 (*strongly agree*). A sample item from this scale is “People cannot be blamed for doing things that are technically wrong when all their friends are doing it too.”

The two dependent variables considered in this report (intervention likelihood and degree of influence of others) were measured using the *Organizational Scenarios Survey*. This survey was developed to assess how people navigate workplace situations in which they witness practices or actions that may result in issues like inefficiencies, inequities, quality problems, or ethical concerns (Hartman et al., 2023). Participants were presented with four short scenarios depicting workplace situations, each highlighting either an ethical problem or an operational inefficiency. Within each scenario, the number of bystanders or individuals present varied from one to 10. The four scenarios are provided in the Supplementary material, Appendix B. Participants were asked to rate their likelihood of taking action to address the situation (*Intervention Likelihood*) on a scale from 1 to 4, where 1 represents “not at all likely” and 4 represents “very likely.” Additionally, they were asked to estimate the degree of influence, if any, that the presence of others had on their decision (*Degree of Influence*) from 1, “not at all” to 4 “a great extent.”

### Statistical methodology

To test whether moral disengagement moderates the differences between autistic and nonautistic employees regarding susceptibility to the bystander effect, we conducted the following steps: (1) We first conducted step-wise backward regression of mixed effects models which included potential confounding factors (e.g., age and sex) in order to determine whether removing the variability explained by these potential confounders worsens the model, i.e., explains significantly less of the variance. (2) We then conducted analyses of variance to test the interaction between moral disengagement and the differences between autistic and nonautistic employees with respect to intervention likelihood and influence of others. (3) If the interaction was not significant, we conducted analyses of variance to test the main effects of moral disengagement within each group. Degrees of freedom vary because these steps were conducted on optimized models that differed in how many potential confounding variables were included in the best fit regression model. The regression model results and ANOVA summary tables are displayed in the Appendix C; Supplementary Tables S1–S7.

## Results

The aim of this study was to determine whether differences between autistic and nonautistic employees with regards to (1) likelihood they would voice concerns about organizational dysfunctions, and (2) degree to which they were influenced by the presence of others when deciding to intervene, are moderated by individual differences in moral disengagement. As predicted, moral disengagement scores were significantly lower among autistic (mean = 1.4) than nonautistic participants (mean = 1.9), *F* (1, 64) = 17.114, *p* = 0.0001. However, our hypotheses are focused on whether the differences found between autistic and nonautistic employees with respect to intervention likelihood and influence of others are moderated by individual differences in moral disengagement.

### Moral disengagement and intervention likelihood

**Figure 1:**
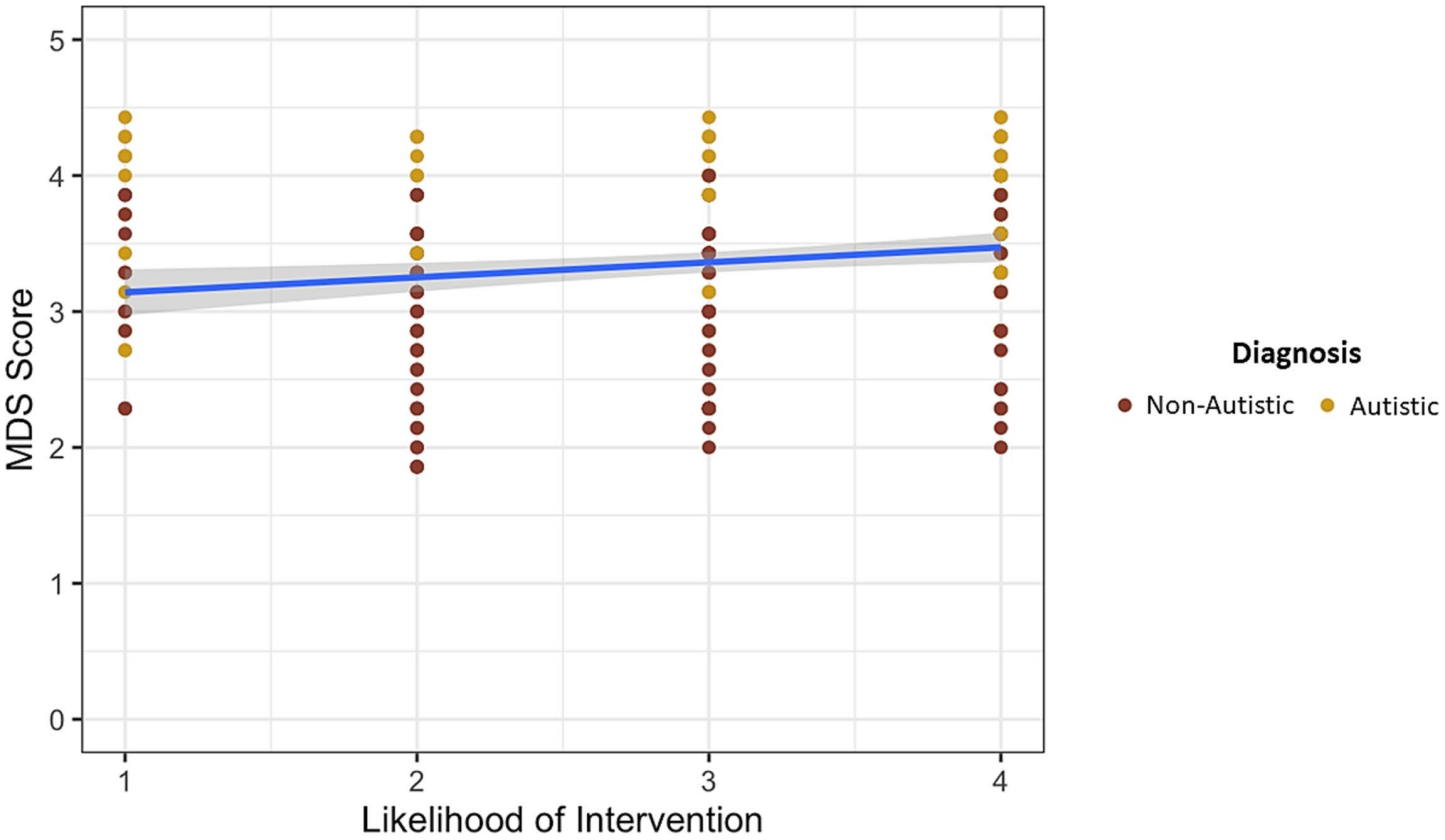
Influence of moral disengagement on likelihood of intervention.

Overall (i.e., autistic and nonautistic participants pooled together), moral disengagement reduces likelihood of intervention, *F* (1, 261) = 19.366, *p* = 0.00002. However, as shown in Figure 1, the influence of moral disengagement in reducing intervention likelihood is not significantly different in the autistic than the nonautistic group, *F* (1, 261) = 0.22, *p* = 0.640.

### Moral disengagement and influence of others

**Figure 2:**
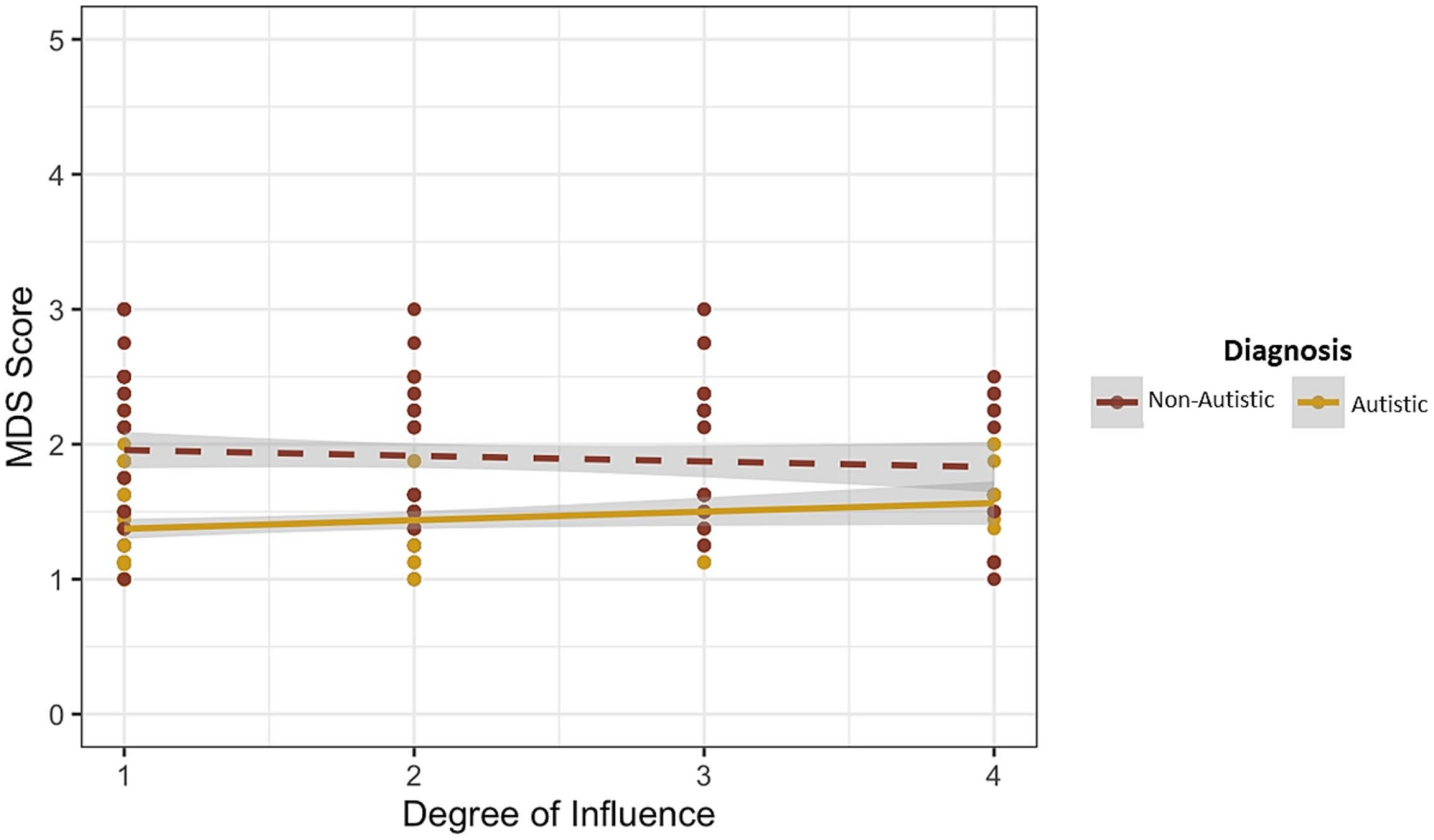
Influence of moral disengagement on degree of influence.

When it comes to estimating the influence of others on the decision to intervene, there is a marginally significant interaction effect. As shown in Figure 2, moral disengagement does differentially affect influence ratings in autistic versus nonautistic participants, *F* (1, 261) = 3.583, *p* = 0.059.

For the autistic group, there is a significant relationship between moral disengagement and degree of influence, *F* (1, 127) = 3.845, *p* = 0.05. However, for the nonautistic group, there is not a significant relationship between moral disengagement and degree of influence, *F* (1, 131) = 0.8671, *p* = 0.354. Although moral disengagement scores are lower among autistic than nonautistic adults, within the autistic group, higher levels of moral disengagement increase the degree to which autistic employees are influenced by others when deciding to intervene. This differential effect of moral disengagement on acknowledging the influence of others is not found with nonautistic employees.

## Discussion

### Theoretical implications

These findings indicate that autistic people are less likely to use moral disengagement strategies in order to justify inappropriate behavior, presumably because autistic individuals exhibit reduced susceptibility to self-serving cognitive distortions and rationalizations commonly observed when most people attempt to explain their actions and decisions. While it has previously been confirmed that moral disengagement is positively related to unethical decisions (e.g., Detert et al., 2008), our findings highlight the bystander effect as one source of potential variation in the pathway linking moral disengagement to unethical behavior. In this study, for both autistic and nonautistic participants, likelihood of intervention decreases as moral disengagement increases (i.e., higher levels of moral disengagement reduced intervention likelihood). The degree to which moral disengagement influenced the decision to intervene did not differ between autistic and nonautistic participants. But levels of moral disengagement did affect the difference between autistic and nonautistic participants when it comes to acknowledging the influence of others on the decision to intervene. Specifically, autistic participants are more likely to acknowledge the influence of others on their decisions as moral disengagement increases whereas this same moderating influence of moral disengagement on acknowledging the influence of others is not evidenced by nonautistic participants. This finding is consistent with numerous reports in the literature of reduced susceptibility to cognitive biases in autism (see Rozenkrantz et al., 2021 for a review). Taken together, these studies suggest that autistic individuals are less susceptible to cognitive biases and exhibit more rational and bias-free processing of information which may be an advantage in ethical decision making.

### Practical implications

Despite the potential organizational benefits of neurodiversity, autistic adults experience rates of unemployment and underemployment as high as 85–90% (Howlin, 2013) and these rates only improve to 75–80% for those with university degrees (Taylor and Selzer, 2011; Hurley-Hanson and Giannantonio, 2016). Many employment practices in the workplace represent potential barriers to hiring, onboarding, and managing neurodiverse employees. For example, hiring assessments may be biased against autistic people (Reilly et al., 2006). Future research should consider the impact of selection practices on the ratio of autistic job applicants hired compared with nonautistic job applicants hired. If the “four-fifths rule” is violated, meaning that the selection ratio for autistic job applicants is less than 80%, or four-fifths, that of nonautistic applicants, then adverse impact has occurred (Roth et al., 2006). Recently, for example, investigators have begun to explore the use of game-based assessments as a way of assessing candidate ability without disadvantaging autistic candidates (e.g., Willis et al., 2021).

Even if they do get hired, there is a need to foster an environment that accepts neurodiversity in the workplace. Challenges include negative attitudes or stereotypes about customizing standardized jobs or tailoring working conditions to accommodate special needs in the workplace (Solomon, 2020). Recently, for example, it has been reported that managers and coworkers are more willing to support workplace accommodations for autistic employees when they have been educated about the myths and realities of attitudes toward autistic people (Khalifa et al., 2020). Finally, future work should consider what organizational systems need to be implemented in order to safeguard autistic employees from potential negative consequences when raising concerns, i.e., “whistleblowing” (Nicholls et al., 2021). Like the metaphor of the canary in the coal mine, while the increased likelihood of reporting organizational dysfunctions may be beneficial to the organization, it is essential to support and applaud these autistic whistleblowers instead of penalizing them.

### Limitations of the research

Limitations include a relatively small sample size, comparison groups (autistic and nonautistic) were not matched in terms of age and sex, and measurement of beliefs or intentions instead of actual behavior. Future studies exploring these effects should employ participant recruitment strategies that ensure a more precise match between autistic and nonautistic groups on key demographic variables. Nonetheless, there is a dearth of research on the potential workplace advantages of autism (see Bury et al., 2020, for a review). Thus, the present research contributes to a growing body of literature focusing on the organizational benefits of neurodiversity (e.g., Austin and Pisano, 2017; Cope and Remington, 2021).

## Conclusion

In summary, these findings suggest that autistic adults are not just more likely to intervene when they witness dysfunction or misconduct in an organizational context; they are also less likely to engage in unethical behavior in general due to lower levels of moral disengagement. As a result of their lower level of moral disengagement, autistic employees are less prone to false beliefs about whether they were influenced by the presence of others when deciding to intervene. Accordingly, the reduced susceptibility to the bystander effect evidenced by autistic adults may be accounted for, in part, by their reduced susceptibility to false beliefs about the influence of situational factors, e.g., the number of other people present, on their decisions and actions.

## Data availability statement

The original contributions presented in the study are included in the article/Supplementary material, further inquiries can be directed to the corresponding author.

## Ethics statement

The studies involving humans were approved by Human Participants Review Sub-Committe, Office of Research Ethics, York University. The studies were conducted in accordance with the local legislation and institutional requirements. The participants provided their written informed consent to participate in this study.

## Author contributions

LH: Conceptualization, Data curation, Formal analysis, Methodology, Writing – original draft, Writing – review & editing. BH: Conceptualization, Project administration, Resources, Supervision, Writing – review & editing.
