# Supplementary material for: An ethical advantage of autistic employees in the workplace
Source: Front Psychol. 2024 Mar 14;15:1364691. doi: 10.3389/fpsyg.2024.1364691 (PMC10973121; doi:10.3389/fpsyg.2024.1364691)

**Supplemental Materials – Appendix A – Participant Information**

**Supplementary Figure 1**: Age of diagnosis for autistic participants


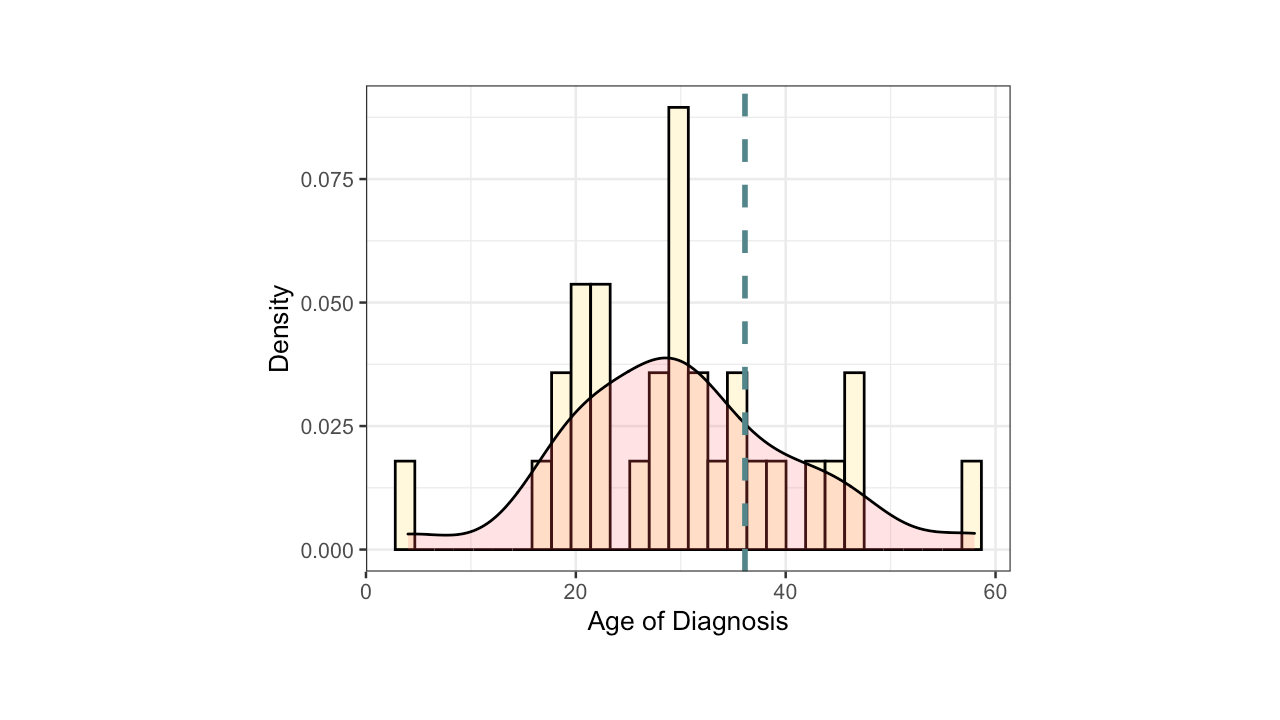


**Supplementary Figure 2**: AQ scores for autistic and non-autistic participants prior to removing outliers.


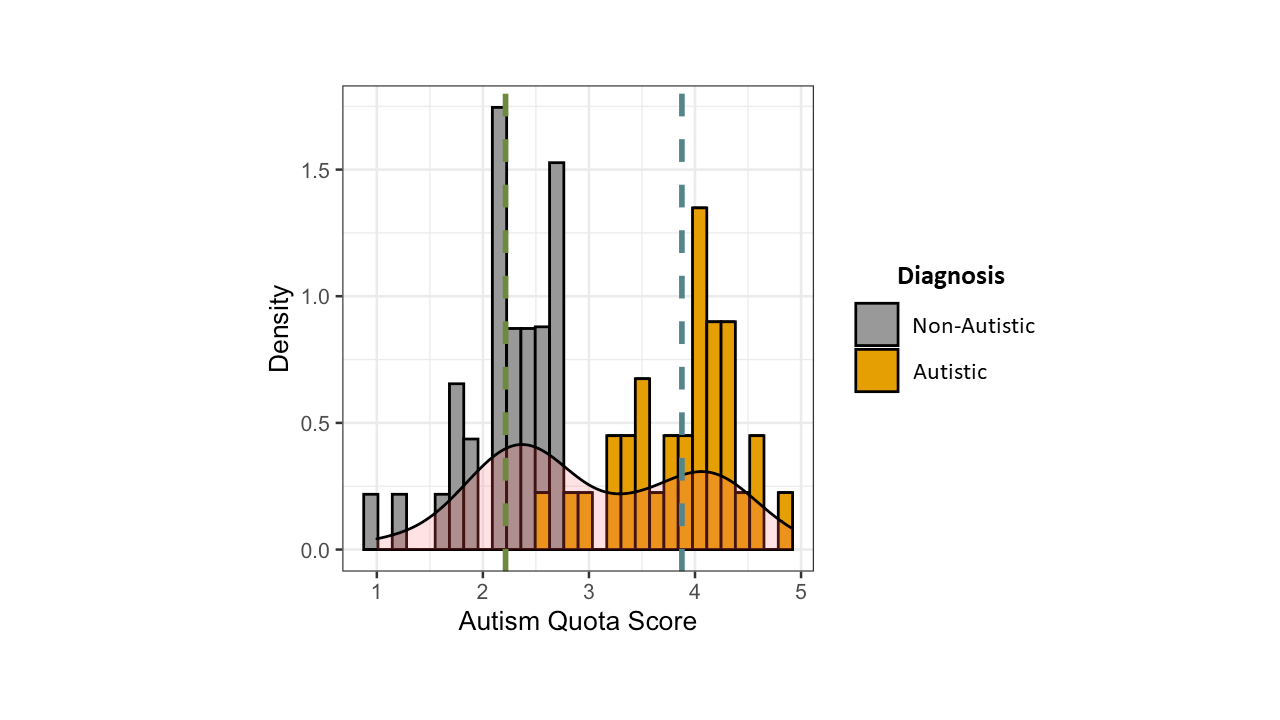


**Supplementary Figure 3:** Employment status of autistic and non-autistic participants.


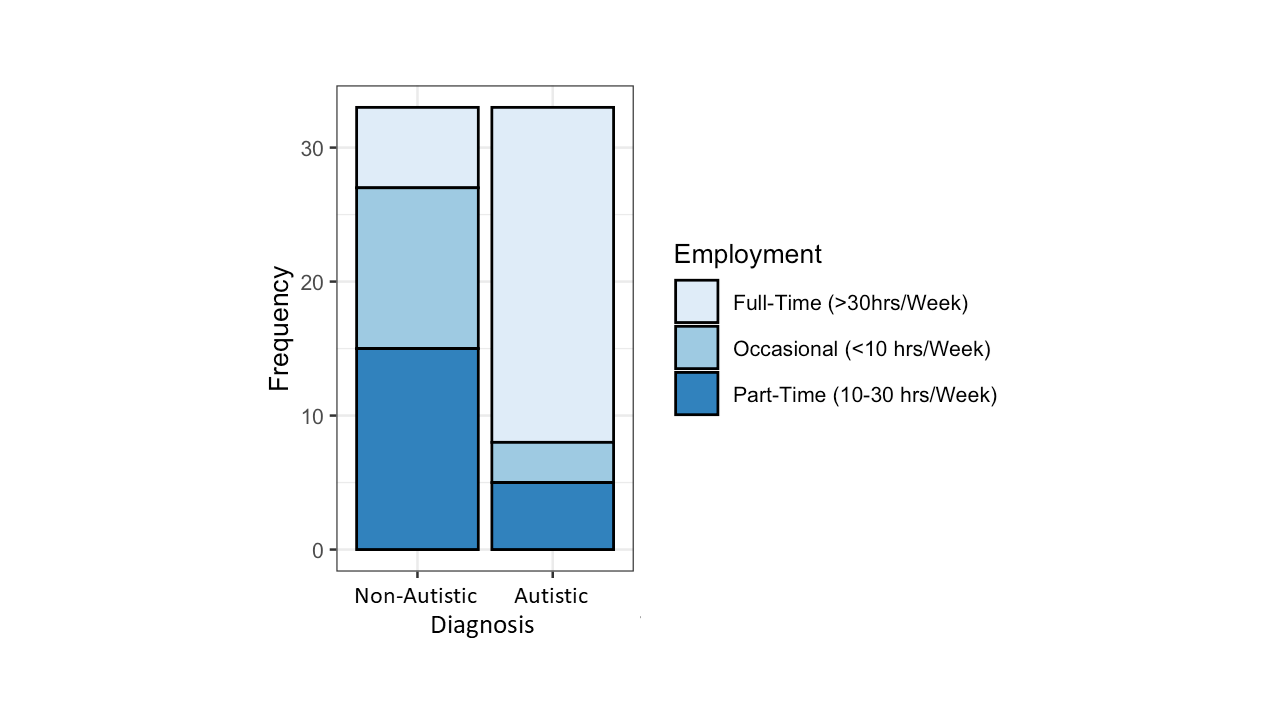


**Supplementary Figure 4.** Type of organization for autistic and non-autistic employees


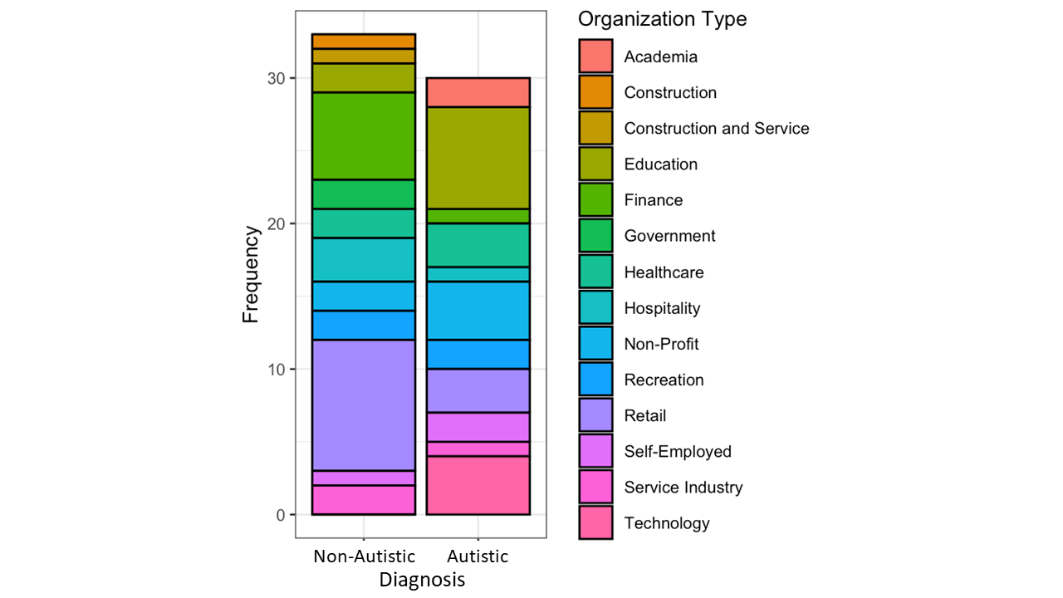


**Supplementary Figure 5.** Length of employment for autistic and non-autistic participants.


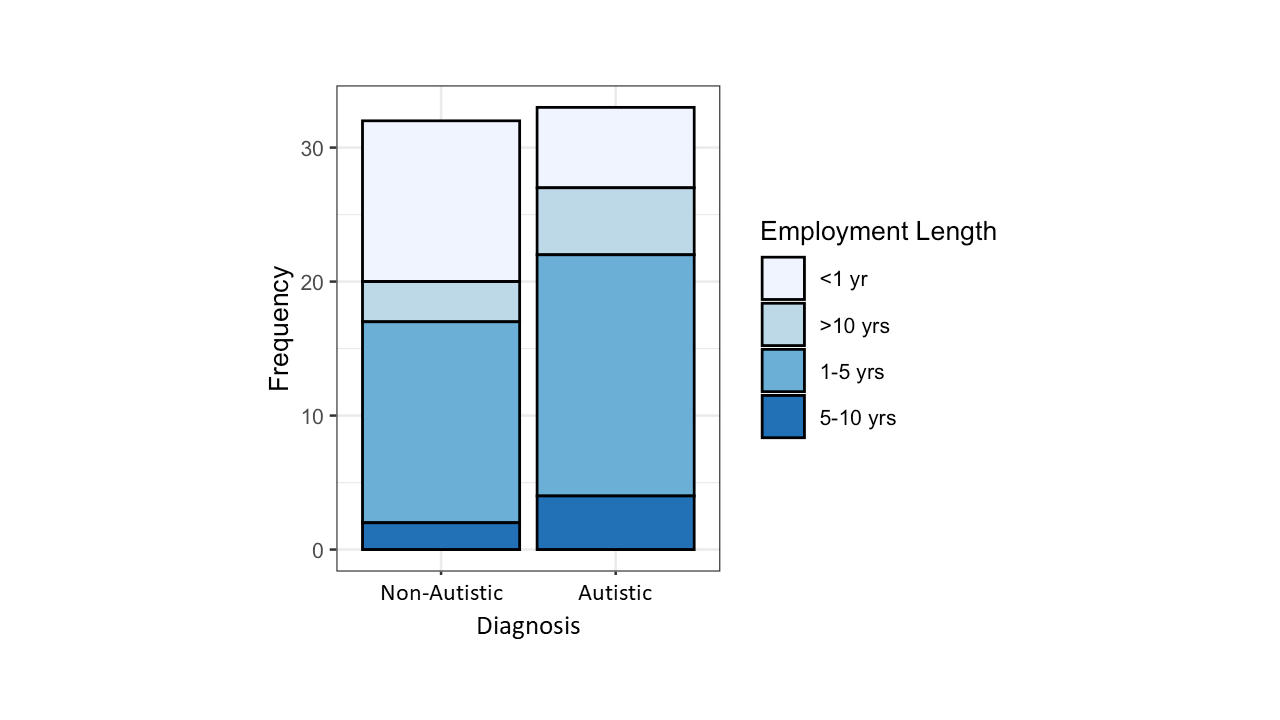


**Supplemental Materials – Appendix B – Organizational Scenarios**

**1 Bystander:** *You are working on a project for a client as part of a team that has been asked to improve the accuracy rate, hence the quality of service, for a specific task. In doing the work, you have been teamed up with a person from the client team to help accomplish this improvement. In the course of doing the work, though, you notice that a major source of inaccuracies, hence low quality of service, are mistakes being made by the client who is your team partner. If you report that back to others, however, it might have negative consequences for your client team partner.*

**2 Bystanders:** *You are working on a software testing team that issues a quality assurance certificate in 8 weeks with 6 weeks consumed by a security process. You and two other members of the team discuss how this security process could be skipped for straightforward “updates” which are unlikely to identify problems. This would reduce the workload of the security process and improve performance to 2 weeks.*

**6 Bystanders:** *You are working on a product development team and during a project meeting with six other team members, the team leader suggests that one of the other team members should try to get confidential information about a competitor’s product by posing as a business school student doing a research project on the competitor’s company and ask for the information.*

**10 Bystanders:** *You are working on a customer support team and receive an e-mail from the Purchasing department that a decision has been made to change the material used in a particular product component to save on production costs. In the e-mail, the Purchasing department Manager advises that customers should not be informed despite potential problems. Ten other team members are copied on the e-mail.*

**Supplemental Materials – Appendix C – Supplemental Data**

**Supplemental Table 1**: Moral Disengagement scores are lower in autistic than nonautistic participants: *F* (1, 64) = 17.114, *p* = 0.0001.


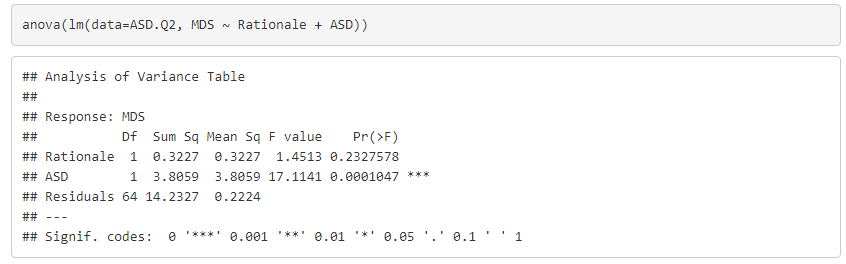


**Supplemental Table 2**: Optimized regression model for Likelihood of Intervention


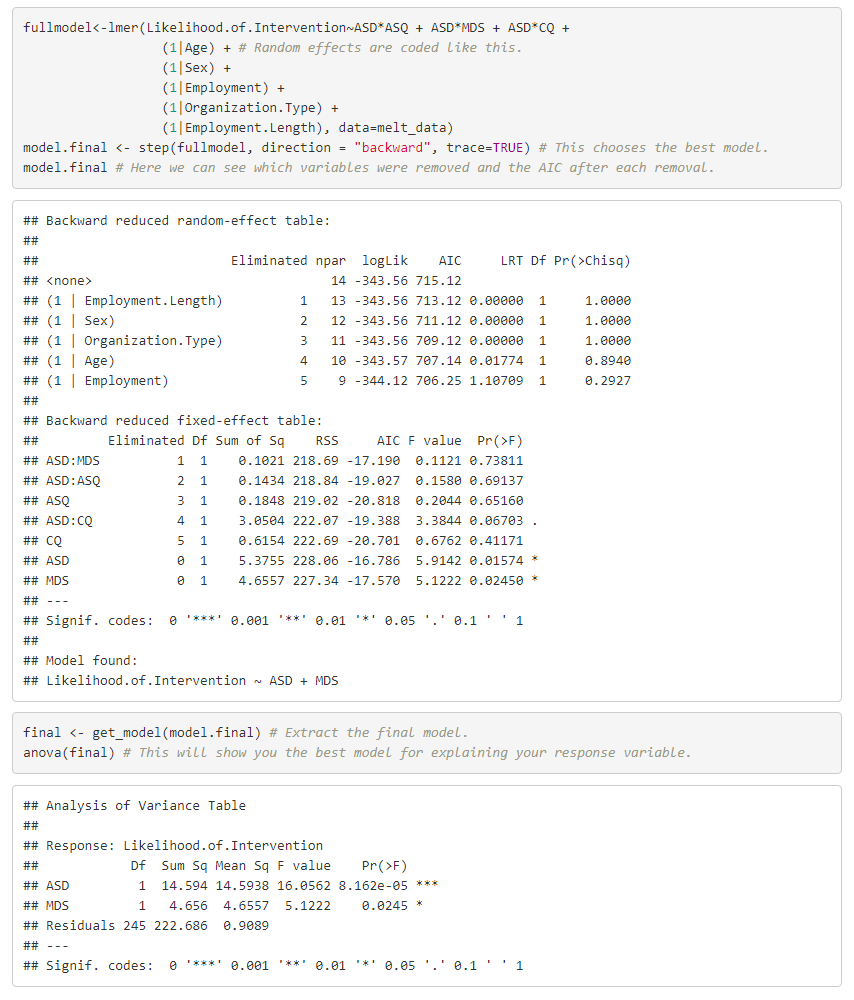


**Supplemental Table 3**: Optimized regression model for Degree of Influence


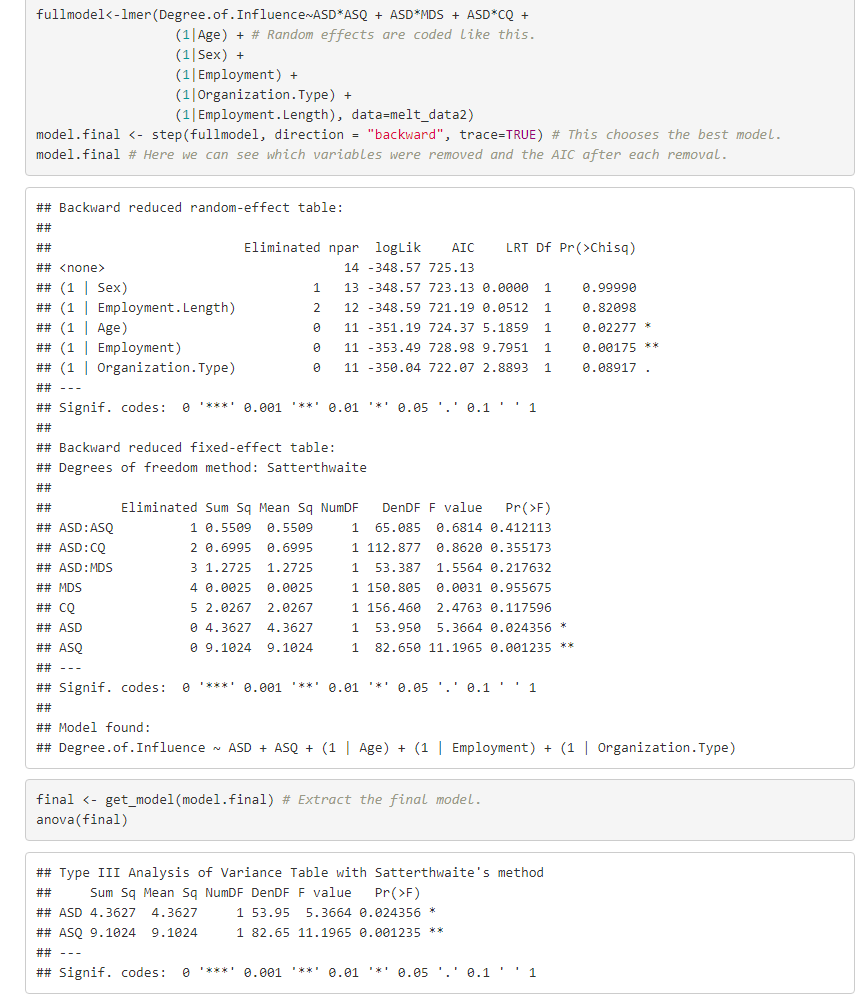


**Supplemental Table 4**: Moral Disengagement reduces Intervention Likelihood for both autistic and nonautistic participants, *F* (1, 261) = 19.366, *p* = 0.00002, but there is not a difference between autistic and nonautistic participants in the extent to which Moral Disengagement moderates Intervention Likelihood, *F* (1, 261) = 0.22*, p* = 0.640.


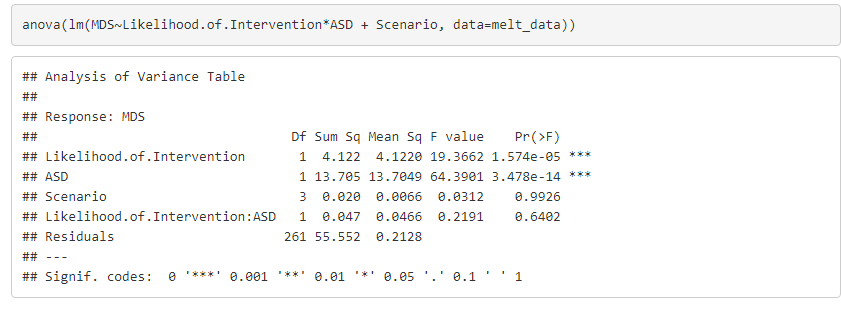


**Supplemental Table 5**: There is a marginally significant difference between autistic and nonautistic participants in the extent to which Moral Disengagement moderates Degree of Influence ratings*, F* (1, 261), = 3.583, *p* = 0.059.


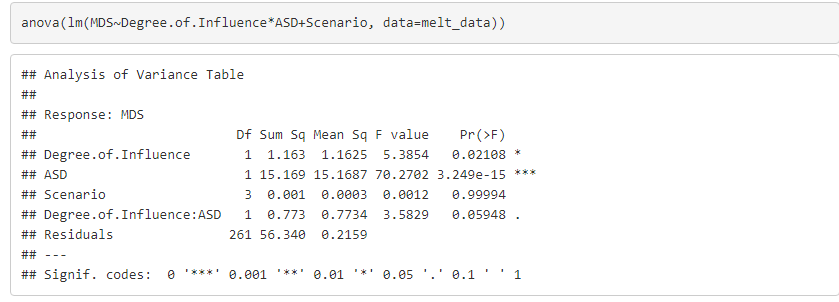


**Supplemental Table 6**: There is a significant difference in the extent to which Moral Disengagement moderates Degree of Influence for autistic participants, *F* (1, 127) = 3.845, *p* = 0.05.


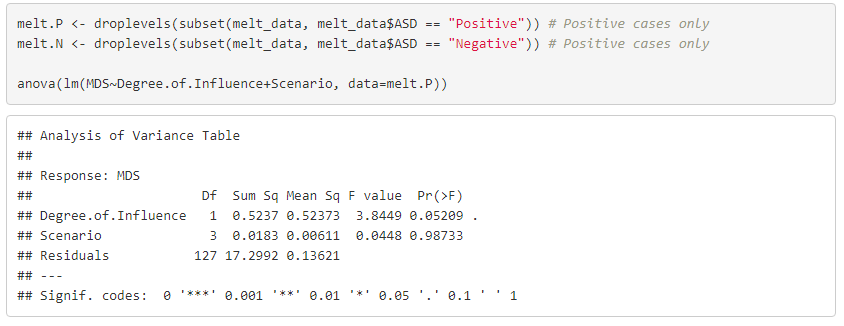


**Supplemental Table 7**: There is not a significant difference in the extent to which Moral Disengagement moderates Degree of Influence for nonautistic participants, *F* (1, 131) = 0.8671, *p* = 0.354.


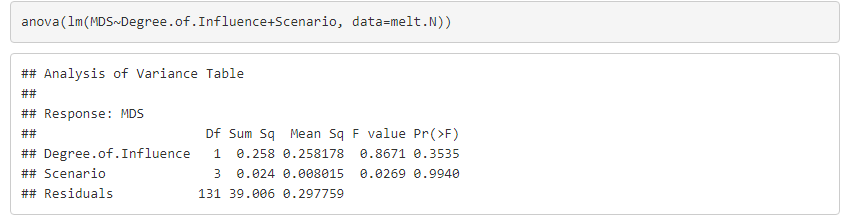

Supplement: Supplementary file 1 [file Data_Sheet_1.docx]
